# No Reduction in Yield of Young Robusta Coffee When Grown under Shade Trees in Ecuadorian Amazonia

**DOI:** 10.3390/life12060807

**Published:** 2022-05-29

**Authors:** Kevin Piato, Cristian Subía, François Lefort, Jimmy Pico, Darío Calderón, Lindsey Norgrove

**Affiliations:** 1 School of Agricultural, Forest and Food Sciences (HAFL), Bern University of Applied Sciences (BFH), Länggasse 85, 3052 Zollikofen, Switzerland; 2 Plants and Pathogens Group, Research Institute Land Nature and Environment, Geneva School of Engineering, Architecture and Landscape (HEPIA), HES-SO University of Applied Sciences and Arts Western Switzerland, Route de Presinge 150, 1254 Jussy, Switzerland; francois.lefort@hesge.ch; 3 National Institute of Agronomical Research (INIAP), Central Experimental Station of Amazonia, km 3 Vía Sacha San Carlos, La Joya de los Sachas 220350, Ecuador; cristian.subia@iniap.gob.ec (C.S.); jimy.pico@iniap.gob.ec (J.P.); dario.calderon@iniap.gob.ec (D.C.)

**Keywords:** *Coffea canephora*, agroforestry, growth, leaf chlorophyll, leaf N, yield, organic versus conventional

## Abstract

Little is known on what impact shade trees have on the physiology of *Coffea canephora* (robusta coffee) under tropical humid conditions. To fill this gap, a field experiment was conducted in the Ecuadorian Amazon to investigate how growth, nutrition (leaf N), phenological state (BBCH-scale) and yield of 5-year-old robusta coffee shrubs are affected by the presence or absence of leguminous trees, the type (organic v conventional) and intensity of management. The experiment was a factorial 5 × 4 design with four cropping systems: intensive conventional (IC), moderate conventional (MC), intensive organic (IO) and low organic (LO), and with five shading systems in a split-plot arrangement: full sun (SUN), both *Erythrina* spp. and *Myroxylon balsamum* (TaE), *M*. *balsamum* (TIM), *E*. spp. (ERY) and *Inga edulis* (GUA). Three monthly assessments were made. Cherry yields of coffee shrubs under moderate shade (c. 25%) were similar to those under high light exposure. Coffee shrubs grown with either *E*. spp. or *I*. *edulis* were taller (+10%) and had higher leaf N concentrations (22%) than those grown without consistent shade. Unless receiving c. 25% of shade, coffee shrubs grown under organic cropping systems showed reduced growth (25%). No correlation was found between height, cherry yield and leaf N. Both shading and cropping systems affected leaf N concentration, also depending on phenological state and yield. Further research is needed to confirm our findings in the long-term as well as to elucidate how leguminous trees may induce physiological responses in robusta coffee under humid tropical conditions.

## 1. Introduction

The main coffee varieties produced and traded are *Coffea arabica* (hereafter “arabica coffee”) and *C*. *canephora* (hereafter “robusta coffee”), which represented 59% and 41% of global coffee production, respectively, in 2019 [1]. Global coffee consumption has been increasing by 1.7% annually since 2015 [1]. Coffee is grown by 25–30 million farmers, of whom 70% are smallholders owning less than 10 ha [2,3]. Since 2010, annual global production of robusta and arabica has increased by 3% and 2%, respectively [1]. Nevertheless, coffee production is threatened by several factors including: (a) climate change which may result in reduction of coffee yield and area climatically suitable for coffee by, e.g., favoring pests and diseases outbreaks [4,5,6,7,8,9]; and, (b) biodiversity loss which affects coffee agroecosystem resilience by reducing, e.g., biocontrol interactions [10,11,12]. 

Both arabica and robusta coffee are grown in Ecuador, which is one of the top 20 coffee-exporting countries, with approximately 6000 tonnes of green coffee produced annually from 2013–2019, although production has decreased by 60% since 1990 [13]. Average coffee yield in Ecuador is low (c. 160 kg/ha in 2018) [13]. Robusta coffee in Ecuador accounts for almost 50% of the total coffee production, mainly produced in the Ecuadorian Amazon Region (EAR), where more than 60% of the farmers cultivate coffee [1,14]. This low productivity has been attributed in the EAR to unsuitable soils, pests and disease losses, and the lack of education and training for farmers [14].

Coffee agroforestry systems (CAS), which include overstorey trees in coffee crops, may provide agricultural, social and environmental benefits [15]. If shade trees are properly selected and managed, CAS can modify microclimate by buffering extreme temperatures, increasing air relative humidity so reducing water losses through both lower soil evaporation and lower crop transpiration [16,17,18]. CAS could therefore constitute an adequate mitigation strategy to counter the detrimental effects of climate change [7,19,20]. Other ecosystem services can be enhanced by CAS such as carbon sequestration [21], soil nutrient availability through complementary partitioning of resources [22,23,24] and biodiversity conservation [25]. However, contrasting shade effects have been reported on coffee growth, productivity, pests and diseases and arabica coffee cup quality [26,27,28,29,30,31]. This might be because shade effects interact with site biophysical parameters [32], diseases [26], shade tree species and their management [16,28,33,34,35], shade level [16,36,37], shade tree age and type of coffee clone [38]. Thus, it is crucial to test interactions among CAS components to highlight the specific conditions under which CAS can be viable. As a general rule, moderate levels of shade (<50%) do not compromise coffee productivity, while heavy shade increases vegetative growth reducing flower bud production, which needs photoperiodism changes and a drop in temperature [39,40]. Nevertheless, lower bud production may be offset by an improved flowering process and fruit set [40,41]. Shade can reduce year-to-year variability in yield, namely for arabica coffee, [42]. Coffee leaf chlorophyll concentration can be modified by shade [43,44], and is positively correlated with leaf nitrogen (N) concentration and can influence coffee aroma [45,46]. N plays a key role in photoinhibition, among others, allowing to reduce occurrence of photodamage and to acclimate the coffee shrub to high irradiance conditions [42,47]. N may also improve net carbon assimilation rate (A) under optimal hydric conditions and stomatal conductance to water vapor (gs) by enhancing carbon isotope discrimination [48,49]. Shade may increase gs, although contrasting effects have been found on A [50,51]. In Ecuador, CAS have already been recognized to play a key role in ensuring food and nutrition security, albeit the increase of productivity of CAS remains a challenge [52].

Although the shade impacts on arabica coffee have been widely assessed, there are few papers dealing with these impacts on robusta coffee and the possible interactive mechanisms behind them [38]. It is important to address shade effects on both arabica and robusta coffee separately, owing to differing ecology [53,54,55] and fertilisation requirements [56]. Furthermore, robusta and arabica coffee may not have the same susceptibility to pests and diseases, which might alter the interactive shade-plant health effect on coffee yield [26,57]. Arabica and robusta coffee species do not have the same pollination type [40] nor the same temporal productivity pattern [58], further underlining the relevance of considering the species as an interactive factor with shade. Treating both species together would have created confounding effect given different responses to shade [54,59,60]. Furthermore, applied work on robusta coffee in Amazonian agroforestry systems is almost non-existent [61]. To help to fill this knowledge gap, we evaluated: (a) how different shading methods combined with different crop management packages (weed, fertilisation and plant health management) may impact physiological responses (height, leaf chlorophyll and yield) of robusta coffee shrubs; (b) if there is any correlation between robusta coffee leaf chlorophyll and N concentration; and (c) to identify the best agroforestry practices for maximising coffee yield.

Our study used three species as shade providers: *Myroxylon balsamum* L. (MB), *Erythrina* spp. (ES) and *Inga edulis* Mart. (IE), all Fabaceae. Litterfall and pruning residues of N_2_-fixing trees might fulfil coffee nutrient requirements [62] by enhancing N mineralization [63] thus benefiting crop nutrition [64,65]. IE is native to Amazonia and provides edible fruit [66,67]. ES, a pantropical legume tree, is widely used as a shelter tree, especially as a green manure [68]. MB is common in tropical forests and could provide a significant income due to its resin being widely used by the pharmaceutical industry [69]. In combination with these shade trees, various cropping systems were assessed, since few authors have tried to link physiological responses of coffee shrubs with both shading management and varying level of farming intensities. Interactions between farming practices (organic and conventional) and shade have been recently reported on coffee morphology [70] and on biogeophysical soil parameters [23], yet these studies dealt exclusively with arabica coffee. Indeed, literature investigating such interactions with robusta coffee is lacking.

**Figure 1:**
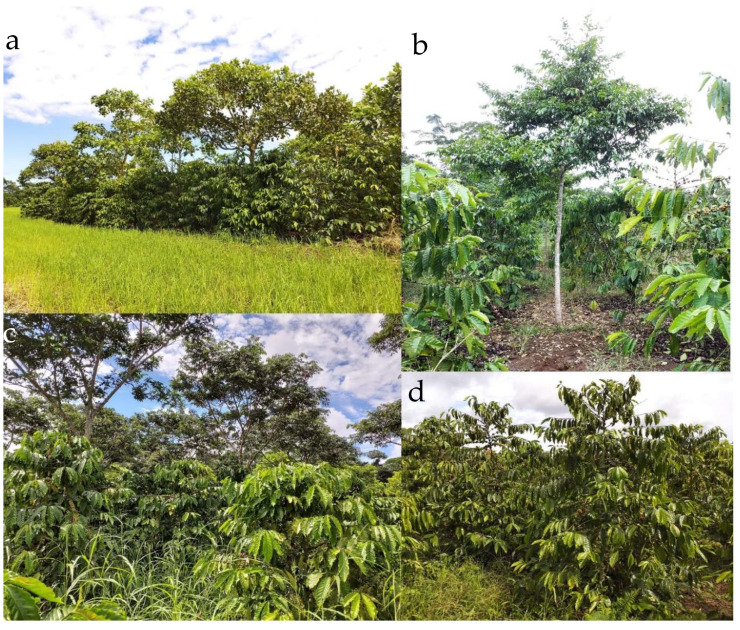
Robusta plants growing under (© Kevin Piato): (**a**) *Erythrina* spp., (**b**) *Myroxylon balsamum*, (**c**) *Inga edulis* and (**d**) full-sun conditions.

We hypothesized that shade could increase robusta coffee growth and leaf chlorophyll concentration, which may be correlated positively with leaf N. Under sub-optimal Amazonian conditions, we hypothesized that robusta coffee yield may not be significantly impacted by shading managements (Figure 1), given that the shade trees were 5 years old and hence provided light shade amount, but rather by cropping systems. We hypothesized that nutrient impacts on physiological responses would be less obvious in plots with higher shade levels, owing to plant growth light-limitation. However, we hypothesized that intensive cropping systems would be associated with increased growth and chlorophyll concentration, especially under shaded conditions given a better nutrient release availability. Furthermore, an association between response variables assessed is expected.

## 2. Materials and Methods

### 2.1. Site Description

The experiment was at the INIAP’s Amazonian Central Station (EECA), La Joya de los Sachas, Orellana, Ecuador (00°21′31.2″ S, 76°52′40.1″ W; 250 m. a. s. l.). Soil is classified as an Andic Dystrudept (30% sand, 27% silt, 43% clay) [71] and the climate as Af (tropical rainforest climate) according to the Köppen–Geiger classification [72]. Vegetation is humid, moist forest [73]. Mean annual temperature is 26.5 °C [74] and mean annual rainfall is 3250 mm [75]. Measurements were made from April–June 2020 with a low rainfall period, followed by a high rainfall period (Table 1; Supplementary Materials, Figures S1 and S2).

### 2.2. Experimental Design

The trial, previously a palm oil plantation, was set up in 2015 in a split-block design with shading system as the main-plot (120 × 36 m) factor and cropping system as the sub-plot (30 × 36 m) factor (*n* = 3). *Coffea canephora* ‘Robusta’ NP-3013 and NP-2024 clones were planted in all plots at a distance of 3 × 2.5 m (1333 shrubs ha^−1^) in 2015. Five shading systems were then applied to the coffee plots and were named as follows: Full-sun (SUN); Timber (TIM); Erythrina (ERY); Guaba (GUA); Timber and Erythrina (TaE). Table 2 gives the main features of each shading system, differing in shade tree species, number of shade tree species, shade tree density, shade amount and pruning regime.

In 2017 and 2018, formative pruning of all shade trees was done. All shading systems except the SUN one also contained *Musa* spp. AAB (plantain) as temporary shade at 333 plants ha^−1^ (5 × 6 m spacing) from 2016 to 2018. Therefore, Table 2 shows shading percentages without banana trees.

Shade %, i.e., the quantity of light (W m^−2^) retained by the shade tree canopy, of the five shading systems was assessed by taking 5 measurements (apex, east, west, north and south) per coffee shrub at 3 m height with a pyranometer Apogee MP-200 under full sunlight conditions. A reference was also taken in full sun next to each coffee shrub. To account for the heterogeneous shade distribution within the plots, zones between shade trees were defined and 3 coffee shrubs per zone were selected (Supplementary Materials, Table S1). Pyranometer measurements were also done at: 900–1030, 1130–1300 and 1400–1530 h. Shade % was thus calculated by taking into account the extent of the different zones encompassing each shading system, the variation of light intensity and sun direction throughout the day. Shade % was calculated with the following formula, for each coffee shrub assessed: shade % = (1 − (average W m^−2^ under shade/W m^−2^ in shade-free)) × (100) and this was used to calculate the mean shade percentage per zone. The weighted average shade % of the shading system was estimated for each time slot as: 

[((average shade % of zone1) × (net area percentage of zone1)) + ((average shade percentage of zone2) × (net area percentage of zone2)) + … + ((average shade percentage of zone18) × (net area percentage of zone18))] × (100).

Finally, the daily average shade % of the plot was estimated by averaging the weighted average shade % of each time slot.

Four cropping systems were also applied to coffee plots: intensive conventional (IC); moderate conventional (MC); intensive organic (IO) and low organic (LO). In conventional plots, synthetic NPK fertiliser was applied 1–2 times per year, yet MC plots received 30% less NPK inputs than the IC ones (Supplementary Materials, Table S2). In organic plots, organic fertilizer (poultry manure) was applied once a year, yet LO plots received 50% less NPK inputs than the IO ones (Supplementary Materials, Table S2). The fertilizer was applied around the coffee stems in all plots. Only coffee shrubs received fertilizer applications.

Weeds were controlled by means of herbicides, with 7 applications per year in IC plots against 5 in MC plots. Furthermore, mechanical weeding with brush-cutters was done, with respectively 4, 5, 9 and 7 interventions in IC, MC, IO and LO plots (Supplementary Materials, Table S3).

Treatments with systemic and organic fungicides, insecticides and *Beauveria* sp. were done (Supplementary Materials, Table S4). IC plots received 2 treatments per year with systemic fungicides, 1 with an organic fungicide and 3 with insecticides mainly to control *Hypothenemus hampei*. MC plots received 1 treatment per year with systemic fungicides, 2 with an organic fungicide and 2 with insecticides. Nevertheless, only an organic fungicide (3 times per year) with *Beauveria* sp. (twice per year) were applied to IO plots. LO plots did not receive any phytosanitary treatment.

Overall, effects of 20 agroforestry treatments (5 shading systems × 4 cropping systems) on robusta coffee height, fresh yield and leaf chlorophyll concentration were assessed.

### 2.3. Measurements

Height was measured once in April 2020 manually from the ground to the apical bud of one randomly selected stem per coffee shrub. The height of 18 coffee shrubs per plot was taken according to a systematic sampling scheme, allowing to include the NP-3013 and NP-2024 robusta clones. This composite sampling avoided the impact of the clone on the variables measured in the study. The same coffee stems were used for all other evaluations. Height was chosen as a suitable vegetative growth measure because (i) crown reduction has never been done on coffee shrubs in the whole trial and (ii) it is well responsive to varying levels of shade and nitrogen inputs [77,78].

Total leaf absolute chlorophyll concentration (TLACC), given in μmol m^−2^, was measured monthly from April to June 2020 with an optical portable chlorophyll meter Apogee MC-100. TLACC measurements were thus in situ and non-destructive. Each coffee stem was divided into 3 equal parts: low, middle and bottom sections. One branch in the middle section was randomly selected, so that 1 branch per selected coffee stem was used. The 4 middle leaves of the selected branch were used for TLACC determination. Two optical measurements per leaf at 1 cm from the main vein and on the middle part of the leaf blade were done. In total, the mean of 8 readings per selected stem from the chlorophyll meter was obtained. Although a significant positive correlation between TLACC and total N concentration (TNC) has already been found [79], we reassessed this correlation, as Apogee MC-100 and SPAD-502 outputs do not follow the same pattern in terms of correlation with TLACC. Thus, 17 TLACC classes ranging from 271 to 780 μmol m^−2^ were defined and 3 randomly selected leaves falling into each class were destructively sampled. TNC, given in %, was analysed by microkjeldahl digestion with sulphuric acid, distilled with sodium hydroxide and collected with boric acid [80]. TNC extractions were conducted by the EECA Soil, Plant and Water Laboratory, La Joya de los Sachas, Orellana, Ecuador. 

Fresh coffee cherry yield (CFY) was assessed monthly from April to June 2020. Prior to harvesting, an extended BBCH-scale describing the phenological stages of Coffea shrubs was used to select the productive coffee stems, i.e., the stems with more than 50% of the fruits (almost) fully ripe, referred to BBCH 85 and 88 growth stages. Coffee fruits of selected stems were then manually harvested and weighted in g. A coffee shrub could have multiple stems.

### 2.4. Statistical Analysis

Statistical analysis was performed with R v. 4.0.2 [81]. Height, TLACC and CFY results were first assessed for residual normality and homogeneity of variance graphically by respectively using residuals versus fits plots and quantile-quantile plots from the lattice package [82]. Variables were transformed when necessary to respect assumption of homoscedasticity and normality before being analysed with linear mixed-effects models from the lme4 package [83]. Height, TLACC and CFY statistical models were, respectively, the following:

lmer(Height_stem ~ Cropping_system × Shading_system + (1|Clone_type) + (1|Stem_nbr) + (1|Block_nbr/Parcel_nbr), data = df_Height_stem);

lmer(TLACC ~ Month × Cropping_system × Shading_system + (1|Stem_nbr) + (1|Block_nbr/Parcel_nbr), data = df_TLACC); lmer(log_CFY ~ Month × Cropping_system × Shading_system + (1|Stem_nbr) + (1|ID) + (1|Block_nbr/Parcel_nbr), data = df_CFY).

Random factors with estimated variance almost null were not included in the models. For the CFY, the ID of the coffee shrub was included as a random factor, owing to possible dependencies between repeated harvests on the same coffee shrub during the 3-month evaluation. Models with varying number of explanatory variables nested with or without interactions were stepwise assessed by selecting the model with the lowest deviance. Lsmeans package [84] was used to get least-squares means, 95% CIs and treatments contrasts with Tukey’s multiple comparisons adjustment. Correlation between height, TLACC and CFY was assessed and plotted through the chart.Correlation function of the package PerformanceAnalytics [85]. Regression analysis were made to assess relation between TLACC and TNC by using ggpubr package [86]. All figures were plotted with the ggplot2 package [87].

## 3. Results

### 3.1. General Results

Cropping system, shading system and month interactively influenced robusta CFY and coffee TLACC (Table 3). Additionally, robusta coffee height was impacted by both cropping system and shading system without any significant interaction.

### 3.2. Coffee Fresh Yield

**Figure 2:**
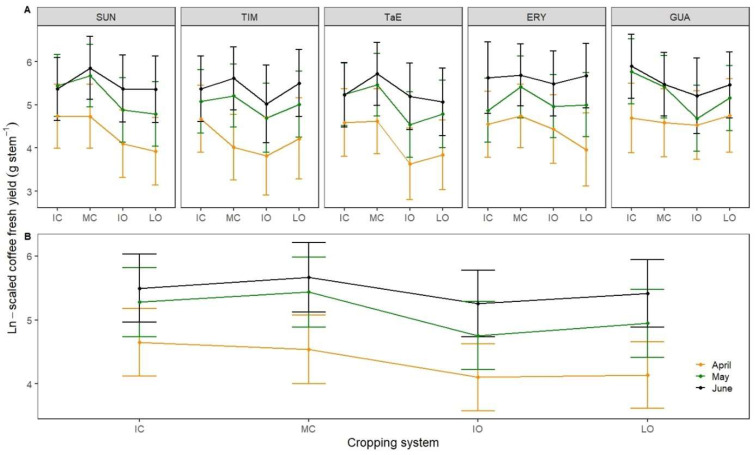
Robusta coffee interaction plots with mean coffee fresh yield as a criterion, cropping system (IC = intensive conventional; MC = moderate conventional; IO = intensive organic; LO = low organic) as predictor and (**A**) both month (April, May, June 2020) and shading system (SUN = full sun; TIM = *Myroxylon balsamum*; TaE = *M*. *balsamum* and *Erythrina* spp.; ERY = *E.* spp.; GUA = *Inga edulis*) as moderators; (**B**) month as moderator. Dots show least-squares means with 95% CIs.

Different combinations of shading and cropping systems resulted in different fruit maturation patterns and yields. (Figure 2A). The TIM and ERY treatments had higher yields in May and June in the moderate conventional than in the intensive conventional (Table 2). The, TaE treatment had higher yields in the low organic than the high ones in April and May (Figure 2A). Yields in moderate conventional were higher than in intensive conventional plots in both May and June (Figure 2B). 

In May, coffee shrubs grown under moderate conventional and full-sun conditions had higher CFY (291 g·stem^−1^) than organic systems, whether full-sun or shaded (−59% with LO × SUN *p* < 0.05; −62% with IO × TIM *p* < 0.05; −68% with IO × TaE *p* < 0.01; −63% with IO × GUA *p* < 0.05). However, plots with an *I. edulis* canopy managed intensively had higher CFY (321 g·stem^−1^) in May compared with those organically managed, whether without shade trees (−63% *p* < 0.05), with *M*. *balsamum* (−66% *p* < 0.05) or both *M*. *balsamum* and *Erythrina* spp. (−71% *p* < 0.01). It is therefore clear that plots without or with shade trees needed intensive management to keep high levels of production. However, plots with a medium level of shade managed intensively did not show a significant decrease in CFY compared to those grown in full-sun conditions (Figure 2A).

**Figure 3:**
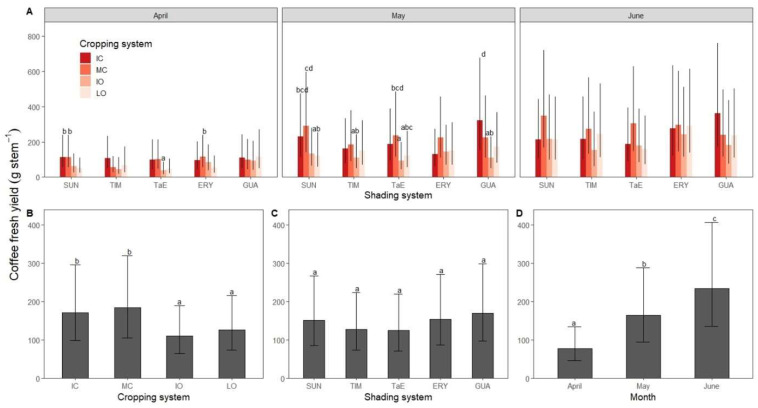
Robusta coffee fresh yield in relation to (**A**) the cropping system (IC = intensive conventional; MC = modera te conventional; IO = intensive organic; LO = low organic) × shading system (SUN = full sun; TIM = *Myroxylon balsamum*; TaE = *M*. *balsamum* and *Erythrina* spp.; ERY = *E*. spp.; GUA = *Inga edulis*) treatments in April, May and June 2020; (**B**) the cropping system; (**C**) the shading system; (**D**) the month. Bars show least-squares means with 95% CIs. Means with the same letter are not significantly different according to Tukey’s test (*p* < 0.05). Absence of letter indicates no significant difference with all other groups.

As the three-way interactions highlighted for the CFY were significant (*p* = 0.011) and homogenous among the treatments, it is worth reporting single factor effects which also influenced robusta coffee yield (Table 3). CFY of conventional plots were greater than organic ones with 110, 125, 183 and 170 g stem^−1^ for IO, LO, MC and IC cropping systems, respectively (Figure 2B). Significant differences in CFY were found between the following cropping systems: IC and IO (*p* < 0.001); IC and LO (*p* < 0.05); MC and IO (*p* < 0.01); MC and LO (*p* < 0.01). CFY did not differ significantly between shading systems (Figure 2C), however, numerically, GUA (170 g stem^−1^) and ERY (154 g stem^−1^) plots yielded more than TaE (125 g stem^−1^) and TIM (128 g stem^−1^) ones with the comparison between TaE and GUA being at *p* = 0.057. CFY per month increased from April to June 2020 (Figure 3D). 

### 3.3. Total Leaf Absolute Chlorophyll Concentration

**Figure 4:**
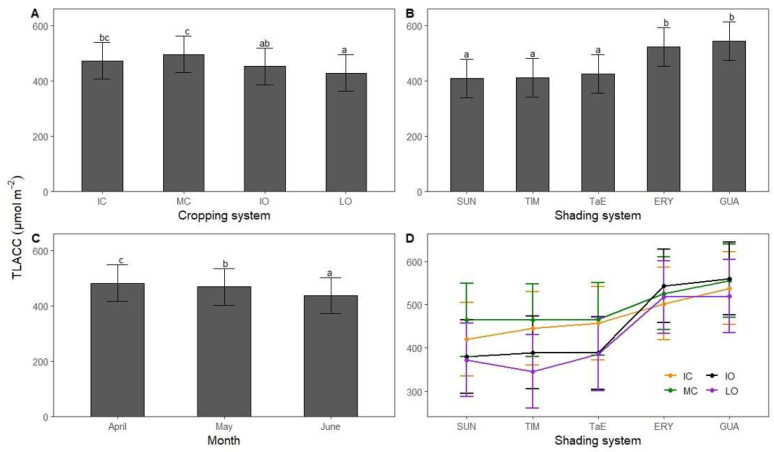
Robusta coffee (**A**) mean total leaf absolute chlorophyll concentration (TLACC) in relation to the cropping system (IC = intensive conventional; MC = moderate conventional; IO = intensive organic; LO = low organic); (**B**) mean TLACC in relation to the shading system (SUN = full sun; TIM = *Myroxylon balsamum*; TaE = *M*. *balsamum* and *Erythrina* spp.; ERY = *E*. spp.; GUA = *Inga edulis*); (**C**) mean TLACC in April, May and June 2020; (**D**) interaction plot with mean TLACC as a criterion, shading system as predictor and cropping system as moderator. Bars show least-squares means with 95% CIs. Means with the same letter are not significantly different according to Tukey’s test (*p* < 0.05).

The effect of shading system on robusta coffee TLACC depended on the cropping system (Figure 3). Interactions were more pronounced for SUN, TIM and TaE shading systems than for ERY and GUA ones. Indeed, TLACC in SUN, TIM and TaE shading systems were higher under conventional than under organic cropping systems. However, in ERY and GUA plots, conventional and organic cropping systems showed approximatively the same TLACC. Post-hoc tests show that conventional cropping systems had higher TLACC than organic ones with 428, 452, 472 and 495 μmol m^−2^ for LO, IO, IC and MC cropping systems, respectively (Figure 4A). Significant differences were found between IC and LO (*p* < 0.001), MC and either IO or LO (*p* < 0.001) cropping systems. Shading systems with reduced shade level (Table 2) had lower TLACC than those with moderate shade level with 409, 411, 424, 522 and 543 μmol m^−2^ for SUN, TIM, TaE, ERY and GUA shading systems, respectively (Figure 4B). Both ERY and GUA shading systems had higher TLACC than SUN, TIM and TaE shading systems (*p* < 0.001). Contrary to the trend observed for CFY, Figure 4C shows that TLACC gradually decreased from April to June 2020 (*p* < 0.001).

### 3.4. Height

**Figure 5:**
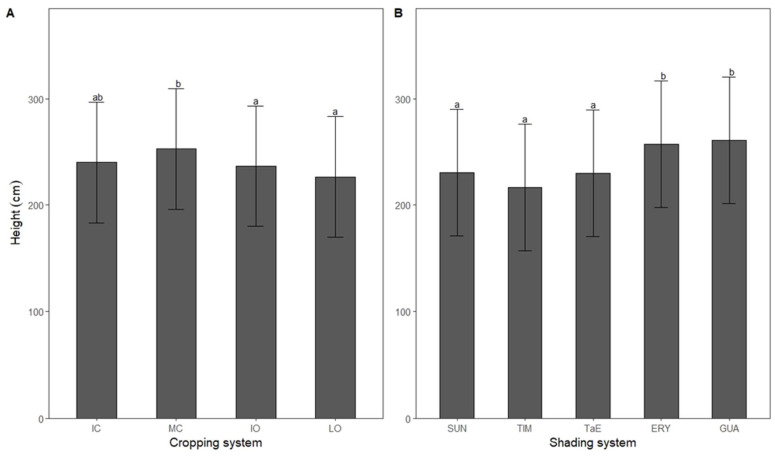
Robusta coffee mean height in relation to (**A**) the cropping system (IC = intensive conventional; MC = moderate conventional; IO = intensive organic; LO = low organic) and (**B**) the shading system (SUN = full sun; TIM = *Myroxylon balsamum*; TaE = *M*. *balsamum* and *Erythrina* spp.; ERY = *E*. spp.; GUA = *Inga edulis*). Bars show least-squares means with 95% CIs. Means with the same letter are not significantly different according to Tukey’s test (*p* < 0.05).

Robusta coffee shrubs under moderate conventional conditions (253 cm) were taller than those under either intensive or low organic conditions (−6% *p* < 0.05). Furthermore, in plots dominated by either *I*. *edulis* (261 cm) or *Erythrina*. spp. (257 cm), coffee shrubs were taller than those without shade shrub (26–30 cm less *p* < 0.001), with *M*. *balsamum* (40–44 cm less *p* < 0.001) or both *M*. *balsamum* and *E*. spp. (27–31 cm less *p* < 0.001), as illustrated in Figure 5B.

### 3.5. Associations between Total Leaf Absolute Chlorophyll Concentration, Coffee Fresh Yield, Height and Leaf Total N Concentration

**Figure 6:**
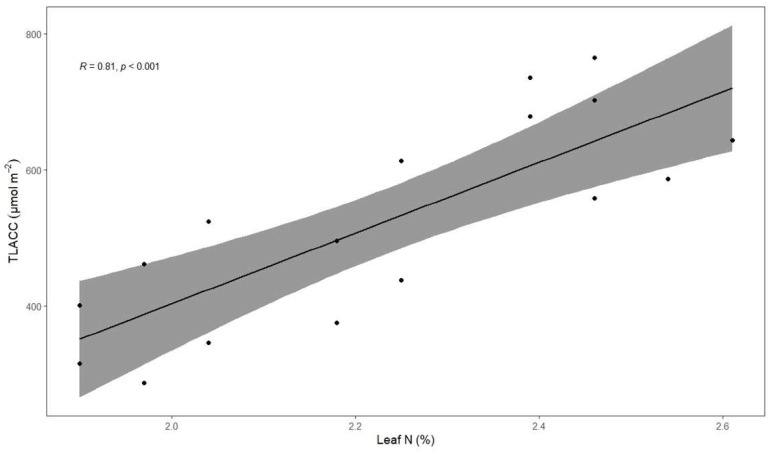
Relation between total leaf absolute chlorophyll concentration (TLACC) and total leaf N (*n* = 17). Line represents regression line fitted. Grey shading shows 95% CI. Pearson correlation coefficient and its *p*-value are also provided.

**Figure 7:**
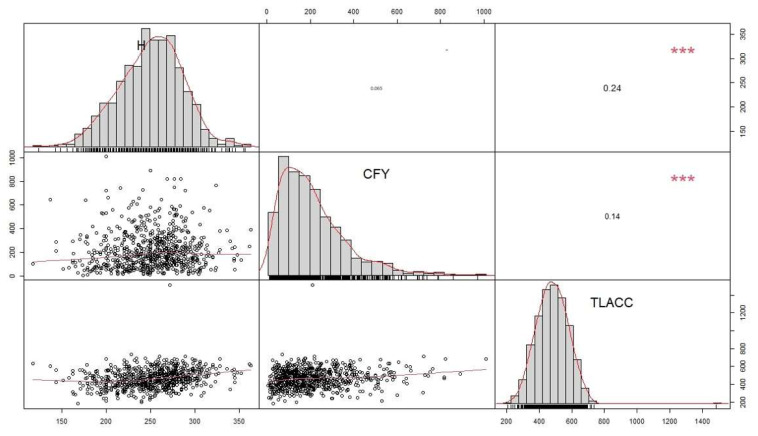
Correlation matrix depicting the correlation between the height (H, *n* = 718), the coffee fresh yield (CFY, *n* = 718) and the total leaf absolute chlorophyll concentration (TLACC, *n* = 718). Across the diagonal histograms with kernel density and rug plots are shown below the diagonal scatter plots with fitted lines. Pearson correlation coefficients with significance level (<0.001 ***) are shown above the diagonal.

TNC was strongly positively correlated with TLACC (*p* < 0.001, Figure 6). TLACC was weakly positively correlated with height (*p* < 0.001), meaning that higher vegetative growth tended to exhibit higher chlorophyll concentration in leaves (Figure 7). However, both TLACC and CFY were not correlated. Therefore, leaf chlorophyll concentration was not correlated with fully ripe cherry yield (BBCH 85 and 88). Additionally, no significant correlation was found between both height and CFY (*p* < 0.05). Consequently, higher leaf N concentration could not be associated with higher fully ripe cherry yield.

## 4. Discussion

### 4.1. Coffee Fresh Yield

Inclusion of shade trees in robusta coffee fields may change fruit-ripening and production patterns, depending on the cropping systems used and the harvest time. High-input cropping systems, i.e., IC and IO, induced earlier and higher yield, most likely because weed, pest and disease pressures were lower. Another explanation would be that increasing weed pressure in low-input systems, regardless of whether organic or conventional, may result in soil water deficit which could fasten cherry maturation [88]. Our results further support that after 3 months of production, robusta CAS, regardless of shade tree used or farming practice, may have the same productivity as robusta coffee grown under full-sun conditions as shade might improve coffee quality, e.g., bean weight [89] by delaying berry ripening by up to 1 month [31] as shade trees might mitigate extreme microclimate variations [90,91]. This finding is in agreement with previous studies [16,27,92]. Nevertheless, considering the production period at an early stage (April or May), robusta coffee grown under either *E*. spp. trees or both *E*. spp. and *M*. *balsamum* trees produced among the highest CFY, when managed conventionally. Schnabel et al. [70] also encountered a similar trend, albeit with high-input systems as they were most productive where the density of shade trees was higher, possibly inducing higher N demand. Table 3 puts forward a significant distinct effect of the shading system on CFY, although post-hoc tests fail to detect consistent differences among shading systems. Our results show that coffee shrubs with moderate shade level (<30%) provided either by *E*. spp. or *I*. *edulis* may yield more than those in full sun conditions, since full-sun coffee shrubs may suffer from dieback associated with heavy bearing causing a low leaf-to-fruit ratio [93]. During the expansion stage, coffee fruits need high quantities of K and N (up to 95% of total N uptake) and are susceptible to N foliar deficiency in case of heavy bearing [39]. Consequently, N deficiency may impair photosynthesis [49]. 

### 4.2. Total Leaf Absolute Chlorophyll Concentration

Robusta coffee TLACC was interactively affected by both shading and cropping systems (Figure 3D). Under light shade or full-sun conditions, TLACC was lower in organic cropping systems than in conventional ones, probably because coffee leaves suffered more from N deficiencies, worsened by heavy bearing. Apparently, the high N requirements were not fulfilled under organic conditions without moderate degree of shading (c. 25%). In our study, we found that N is strongly associated with chlorophyll (Figure 5), which is in agreement with previous studies performed on both arabica coffee and robusta coffee but with a different type of chlorophyll meter [79,94]. Taken all the above into account, TLACC can detect N deficiency. Previous studies have shown that full-sun coffee shrubs had lower N concentrations than shaded ones [89,95], further enhancing the negative effect of organic systems combined with (almost) full-sun conditions on TLACC. Nitrogen deficiency as well as excess light can promote photo-oxidative stress [47], such as H_2_O_2_ production causing stomatal closure and cell membrane damage as well as chlorophyll degradation [96]. This highlights the importance to adapt the nitrogen supply to the intensity of light exposure, meaning that robusta full-sun systems may need more nitrogen than shaded ones to dissipate the excess of energy by the production of both xanthophylls and carotenes [97], as well as produce more antioxidant enzymes to detoxify reactive oxygen species [98]. In our study, TLACC of robusta coffee leaves under either *Erythrina* spp. or *I*. *edulis* trees was higher than that under light shade or no shade, as found in other studies [99,100]. As leaf N concentration have been correlated with soil N concentrations across several species [101], it is likely that *Erythrina* spp. or *I*. *edulis* produced notable biomass resulting in high litterfall inputs [102,103] to the soil which may have improved soil fertility, further enhanced by the ability to fix N. Indeed, it has been demonstrated that *E*. *verna* can fix much N fixation [104] and *E*. spp. can decompose and release N quickly in the soil, owing to low polyphenolic concentrations in the leaves [105]. Therefore, the presence of shade trees providing c. 25% shade might have contributed to sustained continuous nutrient release in robusta CAS. We showed that CFY and TLACC had opposite patterns from April to June (Figure 2D and Figure 3C); higher cherry production resulted in lower chlorophyll and thus N leaf concentration (Figure 5). This might be because late coffee fruit development may induce powerful sink effects which can cause N foliage deficiency, as 95% of total N uptake can be allocated to fruits [39,106]. In contrast, Partelli et al. [107] found higher leaf N concentration during fruit maturation stage, owing to higher N inputs applied which may interact with the phenological stage [108]. However, no correlation was found between CFY and TLACC (Figure 6) most likely because only fully ripe cherries of BBCH 85 and 88 coffee shrubs had been harvested. Therefore, the harvests did not reflect the total coffee production, including non-ripe fruits.

### 4.3. Height

Coffee shrubs were taller when grown under *Erythrina* spp. or *I*. *edulis* shade and this suggested that vegetative growth is more responsive to canopy openness than to competitiveness among aboveground species. Previous investigators found similar results [77,109], even with greater shade percentages, most likely because shade may induce higher light-use efficiency in coffee leaves resulting in increased net primary production [110]. Nevertheless, excessive shade (>70%) may impair plant fitness although this was not the case in our study [26]. Some plants become etiolated under shade [111]. However, results obtained cannot be explained by a concurrent relationship between CFY and height (Figure 5), i.e., reproductive and vegetative development, since no difference was found in yield between shade systems (Figure 2C). Similarly, shade does not affect coffee fruit set [112]. Furthermore, fruiting and leaf production generally do not occur at the same time [58]. Coffee shrubs from MC plots were taller than those from organic plots (Figure 4A) as it is likely that pests and diseases were better controlled there. As the use of pesticides was reduced in MC plots compared to IC plots, it is likely that natural enemies were more active and thus contributed to long-lasting pests and disease control [113].

## 5. Conclusions

Literature on the physiological responses of robusta coffee in agroforestry systems is lacking. During this three-month period, we showed that robusta coffee under moderate shading (c. 25%) yielded as much as when grown under high light exposure, regardless of the level of intensification. Our work highlighted that shade provided by overstorey trees, such as *Erythrina* spp. or *I*. *edulis*, may stimulate vegetative growth of robusta coffee without compromising yield. We presented strong empirical evidence that leaf N concentration is affected by both shading and by the cropping systems. Nitrogen deficiency in low input-systems, whether organic or not, might be avoided by adding leguminous trees with high biomass production. For the first time, we show that the absolute chlorophyll meter (Apogee MC-100) can be used to monitor robusta coffee leaf N concentration under Amazonian conditions. Our study adds to the body of evidence showing that agroforestry systems can improve performance of robusta coffee under sub-optimal conditions. Further research is needed to corroborate our findings in the long-term as well as to elucidate the mechanisms through which leguminous trees may induce physiological responses in robusta coffee under humid tropical conditions.

## Figures and Tables

**Table 1:** Mean monthly daily max and min air temperatures (°C), total monthly precipitation (mm) and mean monthly relative humidity at 7 a.m., 1 p.m. and 7 p.m. (%) from April–June 2020 at the EECA weather station in La Joya de los Sachas, Orellana, Ecuador. Adapted with permission from Ref. [76]. 2020. INAMHI.

	Total Precipitation (mm)	Mean Daily Temperature (°C)		Relative Humidity (%)		
Month		Max	Min	7 a.m.	1 p.m.	7 p.m.
April	86	33	19	97.5	92	96
May	50	32.5	20.5	97	90	96
June	238.5	31	21.5	97	85	92

**Table 2:** Shade tree species, # of shade tree species, shade tree density, shade amount, frequency of shade tree pruning and pruning type for each shading system applied to coffee plots.

	Shading System				
	SUN	TIM	ERY	GUA	TaE
Shade Tree Species	-	*Myroxylon balsamum*	*Erythrina* spp.	*Inga edulis*	*M. balsamum; E.* spp.
# of shade tree species	0	1	1	1	2
Shade tree density (ha^−1^)	0	83	333	83	83
Shade amount (%)	0	7	25	24	9
# of shade tree pruning (year^−1^)	0	0	1–2	1	1–2
Pruning type	-	-	½ pollarding at 2 m; ½ crown reduction (50%)	Crown thinning	Only *E.* spp. crown reduction (50%)

**Table 3:** Results of ANOVA conducted on coffee fresh yield (CFY), height and total leaf absolute chlorophyll concentration (TLACC) for the 3 explanatory variables, i.e., month (M), cropping system (CS) and shading system (SS). Month factor was not included in the height model, since this response variable was measured once. Effects of random variables are not reported. Analysis of variance was performed with Satterthwaite’s method.

Response Variable	Factor	Sum of sq.	Mean sq.	Num. d.f.	Den. D.f.	F-Value	*p*-Value	
CFY (g)	M	166.6	83.3	2	869.6	277.2	<0.001	***
	CS	11.1	3.7	3	37.9	12.3	<0.001	***
	SS	3.3	0.8	4	38.2	2.8	0.042	*
	M:CS	5.6	1.0	6	857	3.2	0.003	**
	M:SS	3.9	0.5	8	864.9	1.6	0.113	
	CS:SS	4.5	0.4	12	37.1	1.3	0.288	
	M:CS:SS	12.9	0.5	24	846.9	1.8	0.011	*
Height (cm)	CS	19,199	6399.7	3	38.2	7.4	<0.001	***
	SS	63,632	15,908.1	4	38.1	18.4	<0.001	***
	CS:SS	19,941	1661.8	12	38	1.9	0.062	
TLACC (μmol m^−2^)	M	1,167,195	58,3598	2	3133.2	75.8	<0.001	***
	CS	391,475	130,492	3	38.2	16.9	<0.001	***
	SS	2,178,395	544,599	4	38.17	70.7	<0.001	***
	M:CS	59,060	9843	6	3133.2	1.3	0.263	
	M:SS	102,495	12,812	8	3133.2	1.7	0.102	
	CS:SS	300,364	25,030	12	38.03	3.3	0.003	**
	M:CS:SS	126,354	5265	24	3133.2	0.7	0.872	

Significances at 95% with ‘***’ *p* < 0.001, ‘**’ *p* < 0.01, ‘*’ *p* < 0.05.

## Data Availability

The data presented in this study are available on request from the corresponding author.

## Supplementary Materials

The following are available online at https://www.mdpi.com/article/10.3390/life12060807/s1, Figure S1: Mean monthly daily max and min air temperatures (°C), total monthly precipitation (mm) from June 2019 to June 2020 at the EECA weather station in La Joya de los Sachas, Orellana, Ecuador [76]. Figure S2: Mean monthly relative humidity at 7 a.m., 1 p.m. and 7 p.m. (%) from January to June 2020 at the EECA weather station in La Joya de los Sachas, Orellana, Ecuador [76]. Table S1: Defined zones with homogeneous shade, according to distance from coffee shrubs to shelter trees. Table S2: NPK inputs in intensive conventional (IC), moderate conventional (MC), intensive organic (IO) and low organic (LO) cropping systems in 2018 and 2019. Table S3: Number of weeding interventions with concentration of active ingredient in brackets in intensive conventional (IC), moderate conventional (MC), intensive organic (IO) and low organic (LO) cropping systems from 2018 to 2020. Table S4: Number of phytosanitary treatments with systemic fungicides (Sf), organic fungicide (Of), insecticides (In) and *Beauveria* sp. (Bb) in intensive conventional (IC), moderate conventional (MC), intensive organic (IO) and low organic (LO) cropping systems in 2019 and 2020. Table S5: Total aggregated coffee fresh yield (g), number of coffee stems harvested and aggregated coffee fresh yield per stem (g) from April to June 2020 in 20 agroforestry treatments combining 4 cropping systems (IC = intensive conventional; MC = moderate conventional; IO = intensive organic; LO = low organic) and 5 shading systems (SUN = full sun; TIM = *Myroxylon balsamum*; TaE = *M*. *balsamum* and *Erythrina* spp.; ERY = *E*. spp.; GUA = *Inga edulis*) located in La Joya de los Sachas, Orellana, Ecuador.
