# Supplementary material for: No Reduction in Yield of Young Robusta Coffee When Grown under Shade Trees in Ecuadorian Amazonia
Source: Life (Basel). 2022 May 29;12(6):807. doi: 10.3390/life12060807 (PMC9224700; doi:10.3390/life12060807)
Supplement: Supplementary file 1 [file life-12-00807-s001.zip › life-1730680-supplementary.pdf]

Supporting Information for: No Reduction in Yield of Young Robusta Coffee When Grown Uunder Shade Trees in Ecuadorian Amazonia

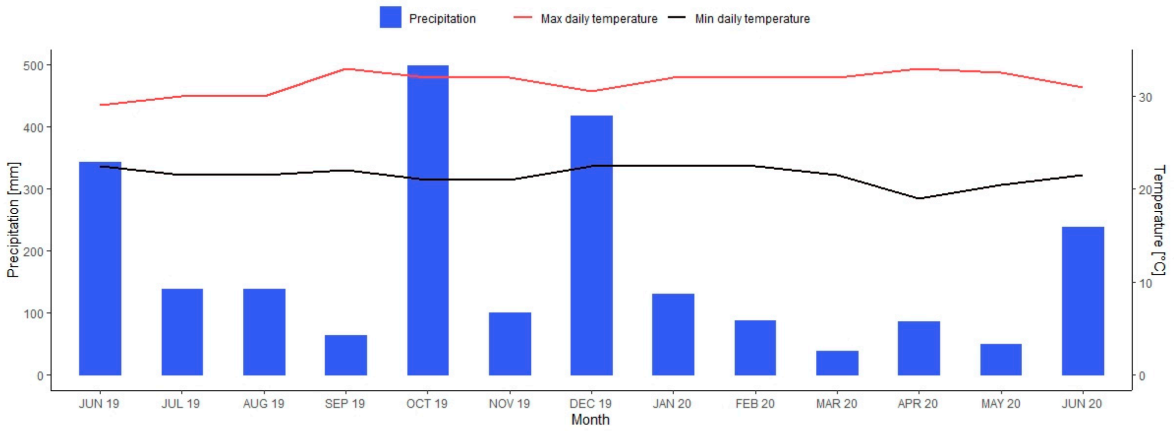

Figure S1. Mean monthly daily max and min air temperatures (°C), total monthly precipitation (mm) from June 2019 to June 2020 at the EECA weather station in La Joya de los Sachas, Orellana, Ecuador [76].

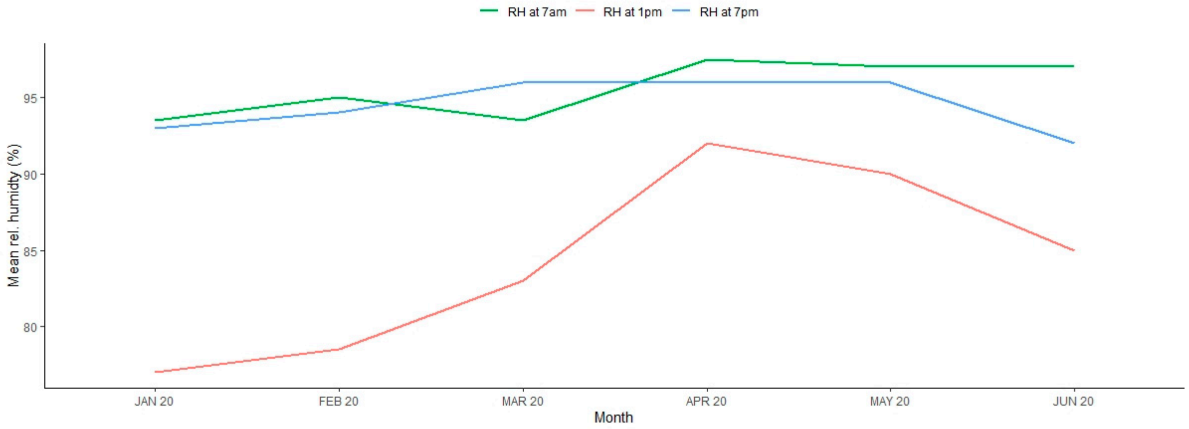

Figure S2. Mean monthly relative humidity at 7am, 1pm and 7pm (%) from January to June 2020 at the EECA weather station in La Joya de los Sachas, Orellana, Ecuador [76].

Table S1. Defined zones with homogeneous shade, according to distance from coffee shrubs to shelter trees.

**Code of zone**	**Name of zone**	**Shading method**	**Net area %**	**Distance between coffee shrubs and shelter trees (m)**
$zone_1$	SUN	SUN	100	-
$zone_2$	$MB_{1.95}$	TIM	11.25	1.95 from *Myroxylon balsamum*
$zone_3$	$MB_{4.04}$	TIM	22.25	4.04 from *M. balsamum*
$zone_4$	$MB_{4.67}$	TIM	22.25	4.67 from *M. balsamum*
$zone_5$	$MB_{5.86}$	TIM	44.25	5.86 from *M. balsamum*
$zone_6$	$IE_{1.95}$	GUA	11.25	1.95 from *Inga edulis*
$zone_7$	$IE_{4.04}$	GUA	22.25	4.04 from *I. edulis*
$zone_8$	$IE_{4.67}$	GUA	22.25	4.67 from *I. edulis*
$zone_9$	$IE_{5.86}$	GUA	44.25	5.86 from *I. edulis*
$zone_{10}$	$ES_{1.95\&4.03P}$	ERY	50	1.95 from *E.* spp.<br>4.03 from *E.* spp. pollarded
$zone_{11}$	$ES_{1.95P\&4.03}$	ERY	50	1.95 from *E.* spp. pollarded<br>4.03 from *E.* spp.
$zone_{12}$	$MB_{1.95}ES_{8.88}$	TaE	11.1	1.95 from *M. balsamum*<br>8.88 from *E.* spp.
$zone_{13}$	$MB_{4.04}ES_{6.43}$	TaE	11.1	4.04 from *M. balsamum*<br>6.43 from *E.* spp.
$zone_{14}$	$MB_{4.67}ES_{7.60}$	TaE	11.1	4.67 from *M. balsamum*<br>7.60 from *E.* spp.
$zone_{15}$	$MB_{7.70}ES_{5.86}$	TaE	22.25	7.70 from *M. balsamum*<br>5.86 from *E.* spp.
$zone_{16}$	$MB_{5.86}ES_{7.70}$	TaE	22.25	5.86 from *M. balsamum*<br>7.70 from *E.* spp.
$zone_{17}$	$MB_{6.43}ES_{4.04}$	TaE	11.1	6.43 from *M. balsamum*<br>4.04 from *E.* spp.
$zone_{18}$	$MB_{7.60}ES_{4.67}$	TaE	11.1	7.60 from *M. balsamum*<br>4.67 from *E.* spp.

Table S2. NPK inputs in intensive conventional (IC), moderate conventional (MC), intensive organic (IO) and low organic (LO) cropping systems in 2018 and 2019.

	Farming practice											
	IC[a]			MC[a]			IO[b]			LO[b]		
	Fertilisation (kg ha^{-1})											
Week # 2018	N	P	K	N	P	K	N	P	K	N	P	K
7	33	16	27	22	11	18						
9							45	26	45	19	11	19
20	20	9	20	13	6	13						
Week # 2019												
8	20	9	20	13	6	13						
22							45	26	45	22	13	22
Sum	73	34	67	48	23	44	90	52	90	41	24	41

[a]KNO_3 13-0-44, KH_2PO_4 0-52-34, $Mg(NO_3)_2$ 11-0-0-16MgO, YaraMila Actyva 27-0-5, YaraMila Hydran 19-4-19-3MgO, NH_4NO_3 34-0-0; Eco Abonaza 3.5-2-3.5 with 70-73% of organic matter (semi-composted bird droppings with sawdust mix)

Table S3. Number of weeding interventions with concentration of active ingredient in brackets in intensive conventional (IC), moderate conventional (MC), intensive organic (IO) and low organic (LO) cropping systems from 2018 to 2020.

	Farming practice							
	IC		MC		IO		LO	
	Weeding method							
Week # 2018	CHEM[a]	MECH[b]	CHEM	MECH	CHEM	MECH	CHEM	MECH
2	✓ (1.8[c])					✓		
3			✓ (1.1[c])					
6						✓		
7			✓ (1[c])					
12						✓		
14	✓ (1.1[c])							
15				✓				✓
19						✓		
20	✓ (1.9[d])							
24	✓ (1.2[d])			✓				✓
26						✓		
27			✓ (1.1[c])					
28	✓ (1.2[d])							
36	✓ (1.2[d])							
37						✓		
40			✓ (1.1[c])					
41								✓
42		✓						✓
44	✓ (0.7[d])					✓		
46				✓				✓
51						✓		
Week # 2019								
4		✓		✓		✓		✓

9		✓		✓		✓		✓
16	✓ (2.2^{c})		✓ (2.2^{c})					
19	✓ (2.2^{c})		✓ (2.2^{c})					
22						✓		✓
25	✓ (2.2^{c})	✓	✓ (2.2^{c})	✓		✓		✓
29		✓		✓		✓		✓
34		✓		✓		✓		✓
35		✓		✓		✓		✓
37	✓ (1.5^{c})		✓ (1.5^{c})					
38	✓ (0.4^{c})		✓ (0.4^{c})					
43		✓		✓		✓		✓
47	✓ (1.5^{c})		✓ (1.5^{c})					
50						✓		✓
Week # 2020								
4	✓ (0.6^{e})		✓ (0.6^{e})					
11						✓		✓
Count	14	8	11	10	0	18	0	15
Count 2018-2019	13	8	10	10	0	17	0	14
# per year	7	4	5	5	0	9	0	7

[a]Chemical weeding; [b]Mechanical weeding; [c]Paraquat ($l\ ha^{-1}$); [d]Goal Tender ($l\ ha^{-1}$); [e]Glyphosate ($kg\ ha^{-1}$)

Table S4. Number of phytosanitary treatments with systemic fungicides (Sf), organic fungicide (Of), insecticides (In) and *Beauveria* sp. (Bb) in intensive conventional (IC), moderate conventional (MC), intensive organic (IO) and low organic (LO) cropping systems in 2019 and 2020.

	Farming practice															
	IC				MC				IO				LO			
	Treatment type															
Week Number 2019	Sf	Of	In	Bb	Sf	Of	In	Bb	Sf	Of	In	Bb	Sf	Of	In	Bb
5	✓[a]		✓[b]		✓[a]		✓[b]			✓[d]						
6												✓[f]				
8																
11	✓[c]		✓[b]			✓[d]	✓[b]			✓[d]						
12												✓[f]				
22						✓[d]				✓[d]						
24												✓[f]				
27		✓[d]	✓[b]													
Week Number 2020																
5	✓[a]		✓[e]		✓[c]		✓[e]			✓[d]						
8	✓[a]		✓[e]			✓[d]	✓[e]			✓[d]						
9												✓[f]				
Count	4	1	5	0	2	3	4	0	0	5	0	4	0	0	0	0
# per year	2	1	3	0	1	2	2	0	0	3	0	2	0	0	0	0

[a]Silvacur 0.45 l ha^{-1} (tebuconozole + triadimesol); [b]Lorsban 0.4 l ha^{-1} (chlorpyrifos); [c]Bankit 0.45 l ha^{-1} (azoxystrobin); [d]Copper oxychloride 50%; [e]Engeo 0.4 l ha^{-1} (lamddacihalotrina + trametoxan); *Beauveria* sp. 10^8 UFC ha^{-1}

Table S5. Total aggregated coffee fresh yield (g), number of coffee stems harvested and aggregated coffee fresh yield per stem (g) from April to June 2020 in 20 agroforestry treatments combining 4 cropping systems (IC=intensive conventional; MC=moderate conventional; IO=intensive organic; LO=low organic) and 5 shading systems (SUN=full sun; TIM=*Myroxylon balsamum*; TaE=*M. balsamum* and *Erythrina* spp.; ERY=*E.* spp.; GUA=*Inga edulis*) located in La Joya de los Sachas, Orellana, Ecuador.

Treatment		Total aggregated yield (g)	# of stems	Aggregated yield (g stem^{-1})
Cropping system	Shading system			
IC	SUN	25441	98	260
IC	TIM	16686	67	249
IC	TaE	14827	74	200
IC	ERY	10585	57	186
IC	GUA	21687	63	344
MC	SUN	29965	101	297
MC	TIM	17108	85	201
MC	TaE	20889	89	235
MC	ERY	30079	108	279
MC	GUA	18784	77	244
IO	SUN	13138	61	215
IO	TIM	5166	36	144
IO	TaE	7295	54	135
IO	ERY	13671	71	193
IO	GUA	6201	48	129
LO	SUN	10031	61	164
LO	TIM	12054	46	262
LO	TaE	7149	50	143
LO	ERY	13174	61	216
LO	GUA	14504	55	264
